# *Notes from the Field:* Impact of the COVID-19 Response on Scale-Up of HIV Viral Load Testing — PEPFAR-Supported Countries, January–June 2020

**DOI:** 10.15585/mmwr.mm7021a3

**Published:** 2021-05-28

**Authors:** Shirley Lee Lecher, Mary Naluguza, Christina Mwangi, Jonathan N’tale, Dianna Edgil, George Alemnji, Heather Alexander

**Affiliations:** ^1^Division of Global HIV & TB, Center for Global Health, CDC; ^2^Country Office, Kampala, Uganda, Division of Global HIV & TB, Center for Global Health, CDC; ^3^U.S. Agency for International Development, Washington, DC; ^4^Office of the Global AIDS Coordinator and Health Diplomacy, U.S. Department of State, Washington, DC.

CDC and the U.S. President’s Emergency Plan for AIDS Relief (PEPFAR) are committed to maintaining an international response to the HIV epidemic even as countries face the challenge of controlling the COVID-19 pandemic (*1*). The Joint United Nations Programme on HIV/AIDS has set the following 95-95-95 targets for HIV infection control by 2030: 1) ensure that 95% of HIV-positive persons are aware of their HIV status, 2) ensure that 95% of these persons receive antiretroviral treatment (ART) and 3) facilitate viral load testing and suppression (viral load ≤1,000 HIV RNA copies per mL of blood) among 95% of persons with HIV infection (*2*). PEPFAR and international donors support 50 countries by investing in diagnostic testing, ART, and viral load testing to monitor treatment outcomes. Recent COVID-19–related stay-at-home orders and travel restrictions have affected essential HIV services worldwide. In the face of these challenges, CDC and PEPFAR are committed to sustaining the momentum necessary to achieve the target goal of facilitating testing and viral suppression among 95% of persons with HIV.

PEPFAR-supported countries,* some with financial resource and workforce limitations, have experienced stay-at-home orders, global flight restrictions, and border closings in response to the COVID-19 pandemic, interrupting supply chains and access to ART (*3*). Health facility mandates have restricted nonessential services, thereby decreasing the availability of ART services and the ability to monitor treatment outcomes with viral load testing (*1*). Early in the COVID-19 pandemic, skilled personnel supporting the HIV epidemic were shifted to the COVID-19 response. Manufacturers of viral load testing platforms developed molecular diagnostic capability for SARS-CoV-2, the virus that causes COVID-19, using the same equipment used for HIV viral load testing. Many laboratory staff members were shifted from molecular testing for HIV to testing for SARS-CoV-2 (*1*). In some countries, laboratory staff members and equipment continue to be shared between the HIV and COVID-19 responses.

Because the limited availability of skilled laboratory staff members and restricted ART access could decrease viral load testing, the effects of the pandemic on viral load testing were examined. The period reviewed was September 2019–June 2020. PEPFAR-supported countries provide quarterly reported data on indicators that monitor the number of patients receiving ART, including viral load testing coverage (the number of ART patients with a documented viral load result within the past 12 months) and HIV viral suppression rates (the proportion of adult and pediatric patients who have been on ART for at least 3 months who have achieved viral suppression). These data were reviewed for viral load testing coverage of ART patients and rates of viral suppression since the COVID-19 pandemic began in March 2020. Data from Uganda are presented as an example.

Viral load testing coverage for all PEPFAR-supported countries was stable at 78% during September–December 2019.^†^ However, viral load testing coverage decreased to 71% during January–March 2020, likely the result of limited access to clinical and laboratory services during the pandemic. After routine services were reinstated (April–June 2020), viral load testing coverage increased to 75%. Among ART patients who received viral load testing, the percentage who were virally suppressed remained stable at 91% during October 2019–March 2020, and at 92% during April–June 2020. This stability in viral load suppression suggests that, although fewer patients on ART were tested (as indicated by decreased viral load testing coverage rates), those who did receive a viral load test had access to ART and were compliant with their ART regimen.

Ugandan government authorities declared a national stay-at-home order on April 1, 2020, in response to the COVID-19 pandemic (*4*). Viral load testing decreased during March–May 2020, with the largest decline occurring late in this period after the beginning of the COVID-19 pandemic (Figure) (*5*). As the government eased restrictions, services were adapted to restore viral load testing. Specific government measures to mitigate the impact of COVID-19 included providing guidance on continuing essential services, increasing the number of viral load specimen pick-ups at testing facilities, expanding collection of dried blood spot specimens (which can be stored and transported without refrigeration) relative to plasma specimens, mobilizing the network of persons with HIV infection to serve as community volunteers to assist others with HIV infection, directly delivering ART to communities, and integrating viral load testing with ART distribution (*4*). This swift response helped restore viral load testing coverage to levels higher than those before the pandemic.

**FIGURE:**
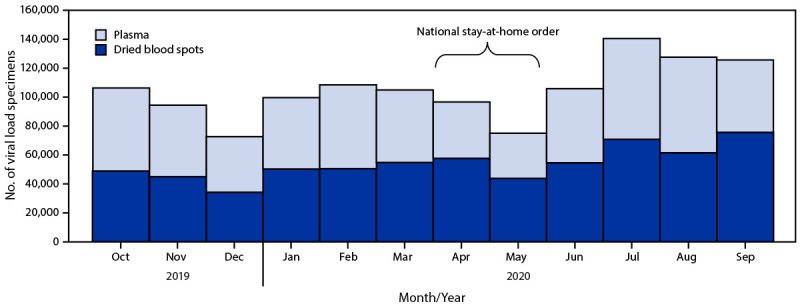
HIV viral load testing, by specimen type — Uganda,*^,^**^†^** October 2019–September 2020 * Data were obtained from the Uganda viral load dashboard (https://vldash.cphluganda.org). ^†^ Stay-at-home order was declared by the government of Uganda on April 1, 2020, and included closure of borders, curfew, restriction of nonessential services, and restriction of public transportation.

During the COVID-19 pandemic, continuation of essential routine care of HIV patients and delivery of routine services will require innovative approaches to reduce the risk for COVID-19 among patients and health care workers. Implementing strategies to return viral load testing services to baseline,^§^ such as clearing testing backlogs to increase the number of persons tested and sustaining services that provide adequate viral load testing to monitor ART patients for treatment success, can maintain HIV control. Access to viral load testing could be facilitated using point-of-care technology for special populations who need expedited testing, including pregnant and breastfeeding women, children with low viral suppression rates, and persons with presumptive ART failure, to prevent clinical deterioration. Despite the challenges of controlling the COVID-19 pandemic, PEPFAR-supported countries should continue advancing toward the 95-95-95 by 2030 goals with expansion of viral load testing for all persons with HIV infection who are receiving ART. Innovative approaches are needed to sustain the global progress made in recent years in response to the HIV epidemic.

## Footnotes

*
https://www.state.gov/where-we-work-pepfar/

† Data for PEPFAR countries were submitted by PEPFAR implementing partners to the U.S. Office of the Global AIDS Coordinator as part of routine program monitoring. The reporting range was July 1, 2019–June 30, 2020.

§ After viral load testing ceased in some countries and decreased in others, specimen backlogs increased during interruption of services. A resumption of testing and return to baseline is necessary before an increase can occur. The 95% is a target to be reached by 2030 as countries continue to scale up HIV viral load testing, which just started in most sub-Saharan countries 2013–2014.
